# Hypoxia triggers the outbreak of infectious spleen and kidney necrosis virus disease through viral hypoxia response elements

**DOI:** 10.1080/21505594.2022.2065950

**Published:** 2022-04-25

**Authors:** Jian He, Yang Yu, Zhi-Min Li, Zhi-Xuan Liu, Shao-Ping Weng, Chang-Jun Guo, Jian-Guo He

**Affiliations:** a State Key Laboratory for Biocontrol, Southern Laboratory of Ocean Science and Engineering, Zhuhai, Guangdong, PR China; b School of Marine Sciences, Sun Yat-sen University, Zhuhai, Guangzhou, PR China; c Guangdong Province Key Laboratory for Aquatic Economic Animals, and Guangdong Provincial Key Laboratory of Marine Resources and Coastal Engineering, Sun Yat-sen University, Guangzhou, PR China

**Keywords:** Iridovirus, ISKNV, hypoxia, hypoxia response elements, HIF pathway

## Abstract

Hypoxia frequently occurs in aquatic environments, especially in aquaculture areas. However, research on the relationship between hypoxic aquatic environments with viral diseases outbreak is limited, and its underlying mechanisms remain elusive. Herein, we demonstrated that hypoxia directly triggers the outbreak of infectious spleen and kidney necrosis virus (ISKNV) disease. Hypoxia or activated hypoxia-inducible factor (HIF) pathway could remarkably increase the levels of viral genomic DNA, titers, and gene expression, indicating that ISKNV can response to hypoxia and HIF pathway. To reveal the mechanism of ISKNV respond to HIF pathway, we identified the viral hypoxia response elements (HREs) in ISKNV genome. Fifteen viral HREs were identified, and four related viral genes responded to the HIF pathway, in which the *hre-orf077r* promoter remarkably responded to the HIF pathway. The level of *orf077r* mRNA dramatically increased after the infected cells were treated with dimethyloxalylglycine (DMOG) or the infected cells/fish subjected to hypoxic conditions, and overexpressed *orf077r* could remarkably increase the ISKNV replication. These finding shows that hypoxic aquatic environments induce the expression of viral genes through the viral HREs to promote ISKNV replication, indicating that viral HREs might be important biomarkers for the evaluation of the sensitivity of aquatic animal viral response to hypoxia stress. Furthermore, the frequencies of viral HREs in 43 species aquatic viral genomes from 16 families were predicted and the results indicate that some aquatic animal viruses, such as *Picornavirdea, Dicistronviridae*, and *Herpesviridae*, may have a high risk to outbreak when the aquatic environment encounters hypoxic stress.

## Graphical abstract

**Figure:**
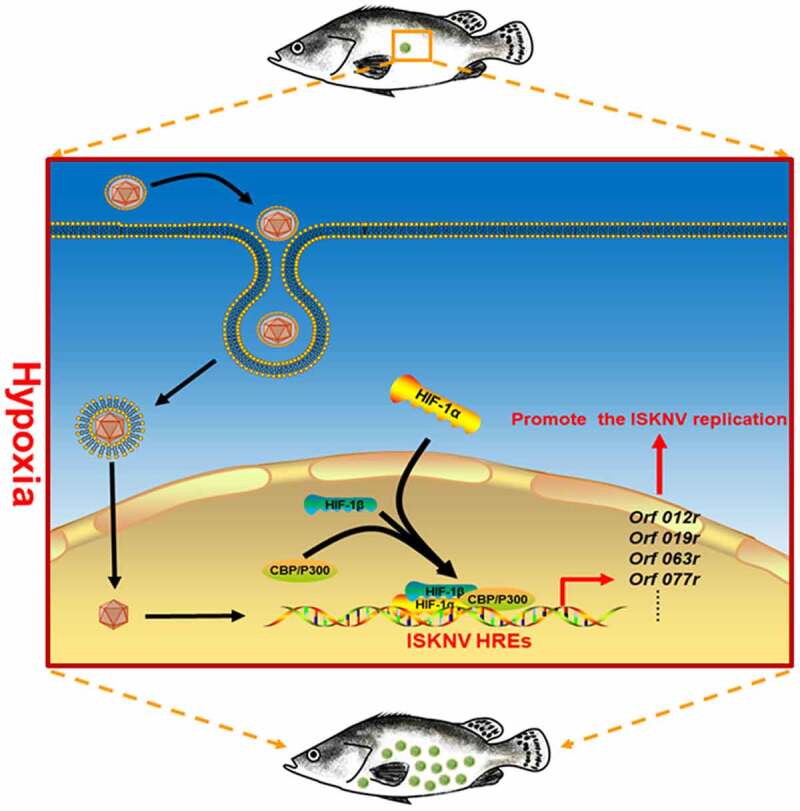

## Introduction

Infectious disease outcomes, outbreak timing, and pathogen invasiveness are shaped by the interactions among hosts, pathogens, and environment [1]. Changes in the environment influence host–pathogen interactions and the emerging/reemerging rate and transmission patterns of pathogens [2,3]. Hypoxia, which refers to the reduction in the normal levels of oxygen, remarkably affect viral propagation [4]. Hypoxia promotes viral replication through various ways. For instance, hypoxia can promote viral replication by upregulating special host gene expression. Hypoxia induces hepatitis C virus (HCV) by increasing the hepatocellular autotaxin expression, which positively regulates HCV replication [5]. Hypoxia enhances the replication of oncolytic herpes simplex virus G207 by increasing the growth arrest and DNA damage-inducible protein 34 expression in U87 human glioma cells [6]. Hypoxia could reprogram the host metabolism to improve viral replication. Physiological hypoxia enhances human T-lymphotropic virus-1 plus-strand transcription by influencing glucose metabolism [7]. Moreover, hypoxia could promote the viral gene expression. Hypoxia promotes the expression of Kaposi’s sarcoma-associated herpesvirus (KSHV) viral genes, including processivity factor 8, glycoprotein 8.1, and viral interleukin 6 to activate viral replication [8]. Under hypoxia, the expression of the Epstein-Barr virus (EBV) immediate-early protein Zta was increased and triggered EBV lytic replication [9].

The HIF pathway is one of the most important oxygen-sensing regulatory networks that help organisms tolerate hypoxia [10]. The transcription factor of the HIF pathway is HIF complex, which is a heterodimer consisting of either HIF-1α or HIF-2α and HIF-1β subunits [11]. Upon dimerization, the HIF transcription factor is located in the nucleus, binds to E-box-like hypoxia response elements (HREs, 5‘[A/G]CGTG-3’) of the promoter region, and then recruits the p300/CBP protein to initiate the transcription of downstream genes [12]. The HIF pathway is involved in various diseases from cancer to infection [13]. During virus–host interactions, the HIF-pathway is an important target manipulated by various viruses [14]. In mammals, viruses hijack the HIF pathway to facilitate their infection. For instance, lytic EBV infection is triggered by the activated HIF pathway. HIF-1α directly binds the promoter of the EBV immediate-early BZLF1 gene, which contains an HRE element, and induces the BZLF1 gene expression [15]. John Cunningham virus (JCV)-infected glial cells activate the HIF pathway, and HIF-1α binds to the control region of JCV to regulate both its early and late promoters [16]. The long terminal repeat (LTR) regions of some human immunodeficiency virus (HIV) strains contain HREs; HIV replication is suppressed when the HIF complex interacts with those HIV-HREs [17]. However, in lower vertebrates, the relationship between the HIF pathway and the outbreak of viral diseases remains unclear.

Hypoxia, in which the amount of dissolved oxygen (DO) ≤2 mg liter^−1^ [18], frequently occurs in aquatic environments, and it negatively affects diverse aquatic ecosystems, ranging from vast oxygen-minimum zones in oceans to small ponds less than a hectare in size [19]. Hypoxia frequently occurs and persists in aquaculture areas because of intensive culture conditions [20]. However, limited research has focused on how hypoxic aquatic environments induce the outbreak of infectious diseases, and the roles of hypoxic environments in the outbreak of aquatic infectious diseases, especially viral diseases, have not been experimentally determined. Infectious spleen and kidney necrosis virus (ISKNV) belong to the species of the family *Iridoviridae* and have been detected in over 50 fish species with high mortality rates, thus seriously threatening the aquaculture industry and aquatic ecosystems [21,22]. In the present work, the interaction of ISKNV and its host mandarin fish (*Siniperca chuatsi*) under hypoxia was employed as model to investigate the relationship of hypoxic aquatic environment and viral disease outbreak. Results showed that the viral HREs in ISKNV genome responded to hypoxic stress and then promoted the ISKNV disease outbreak. Therefore, aquatic viral HREs might be used as a molecular marker to evaluate the risk of aquatic viral disease outbreaks under hypoxia. Our work offers guidance to the prediction of aquatic disease outbreaks and the development of disease-resistant varieties, drug targets, and aquaculture models.

## Materials and methods

### Fish

A total of 800 mandarin fish (*S. chuatsi*) with body weight of 50–100 g was obtained from a fish farm in Nanhai, Guangdong province, PR China. All animal experiments were permitted by the ethics committee of Sun Yat-sen University (No. 2019121705).

### Cell culture

Mandarin fish fry (MFF-1) cells were cultured using Dulbecco’s modified Eagle medium (DMEM, GIBCO, USA) containing 10% fetal bovine serum (Hyclone, USA) at 27°C and 5% CO_2_ [23].

### Viral infection

The ISKNV strain (NH-2017) was isolated from the spleen of the moribund mandarin fish with symptoms of ISKNV infection in Nanhai fish farm, which was provided by associate Prof. Shao-Ping Weng in 2017 [24]. MFF-1 cells were seeded in plates for 24 h, and then exposed to ISKNV at 1 µl 1.196 × 10^9^ TCID_50_/mL dilution in 1 mL of culture medium. After incubation for 4 h, the ISKNV was removed, and a new culture medium was added. The mandarin fish samples were intraperitoneally injected 100 µl ISKNV with 1.196 × 10^7^ TCID_50_/mL

### Hypoxia treatment

For the treatment group, cells were cultured in the hypoxic cell incubator (Smartor 118 pro, Huayiningchuang Co., Ltd, China) under 3% O_2_. For the control group, cells were cultured in the normoxia (21% O_2_) cell incubator (Forma 2, Thermo Fisher Scientific, USA). The mandarin fish were cultured in hypoxic aquarium with 2 mg/L DO (Figure S1); for control group, the fish samples were cultured under normoxia with 7 mg/L DO. Each group with 30 fish samples for experiment.

### Histology analysis

Spleen samples were separated from the ISKNV-infected mandarin fish and placed into 10% formalin fixed. After 12 h, the samples were dehydrated using ethanol. Then, the samples were embedded using paraffin and cut into 4 μm-thick sections. The sections were stained with hematoxylin and eosin and examined under a microscope.

### Quantification of viral genomic copies by absolute quantitative real-time PCR (qPCR)

DNA was extracted from the infected mandarin fish and MFF-1 cells by using DNeasy Blood and Tissue Kit (Qiagen, Germany). The amount of viral major capsid protein (*isknv-mcp*) gene copies was used to determine the level of ISKNV genomic copies. The number of *isknv-mcp* gene copies was determined using the standard curve method of qPCR. The primers are listed in Table S2. The PCR reaction mixture contained 5 μl of 2 × SYBR Premix Ex Taq (TaKaRa, China), 1 μl of DNA template, .2 μl of 10 μM primers, and 3.6 μl of H_2_O. The absolute qPCR conditions were as follows: one cycle at 95°C for 10 s, 40 cycles of 5 s at 95°C 40 s at 60°C and 1 s at 72 °C.

### Quantification of gene expression by quantitative reverse transcription PCR (qRT-PCR)

The RNeasy mini kit (Qiagen, Germany) was used to extract total RNA from the infected mandarin fish and MFF-1 cell samples. PrimeScript RT Reagent kit (TaKaRa, China) was used to reverse transcribe RNA samples to cDNA. qRT-PCR assays were conducted using Roche LightCycler480 thermal cycler (Roche Applied Science, Germany). The primers are listed in Table S2. The reaction volume and parameters of the cycling were the same as those described above.

### TCID_50_ assay

Cells were seeded into 96-well dishes. After growing overnight, cell density exceeded 80%. The original ISKNV samples were diluted using 10^−1^ – 10^−10^ of culture medium. Each concentration gradient of the virus was added into eight wells, and 100 μl of culture medium without virus was placed in eight wells as negative control. Then, the 96-well dishes were incubated at 27°C After 7 days of incubation, the number of positive and negative wells were recorded after 7 days of infection; the TCID_50_ was calculated using Spearman-Karber method [25,26].

### Dual luciferase reporter assays

Cells were seeded into 24-well plates. After 24 h of culture, the cells were co-transfected with the pGL3-ISKNV HREs-luc (.8 µg per well) and pRL-TK (80 ng per well) plasmid. The pRL-TK plasmid was used as an internal control. At 48 h post-transfection, the total cells were harvested and lysed. The Dual Luciferase Reporter Gene Assay Kit (Promega, USA) was used to measure the induction of the reporter genes in the lysate.

### Western blot

The infected cells were harvested and lysed. The lysates were mixed with 5× loading buffer, boiled for 10 min, and subjected to SDS-PAGE for separation. The samples were subsequently transferred on the nitrocellulose membranes. Afterward, the membranes were blocked using a blocking buffer at room temperature for 1 h. Then, the membranes were washed thrice for 5 min each with TBST. Thereafter, the membranes were incubated with anti-VP101R (an ISKNV viral structural protein) antibody (mAb2D8) at room temperature for 2 h, and then washed thrice for 5 min each with TBST. Next, the membranes were incubated with goat anti-mouse IgG HRP conjugate (Promega, USA) at room temperature for 1 h, and then washed thrice for 5 min each with TBST. Finally, protein bands on the membranes were visualized using a high-sig chemiluminescence WB substrate kit (Tanon, China).

### HREs predicted in viral genome and calculated frequencies of viral HREs

The sequences of viral HREs were analyzed using the sequence selected from the TRANSFAC collection. The Gen Bank ID of the virus used in this analysis is listed in Table S1. The frequencies of viral HREs were calculated using the formula below [27]:

$$frequencies\;of\;viral\;HREs=\frac{total\;numbers\;of\;viral\;HREs}{number\;of\;each\;virus\;open\;reading\;frames}$$

### Electrophoretic mobility shift assay (EMSA)

The biotinylated or unbiotinylated oligonucleotides of the putative HRE binding motifs are shown in Figure 5A. All probes were synthesized by Life Technologies (Carlsbad, CA, USA). The synthesized oligonucleotides were diluted to 10 μM, and then annealed to double-stranded probes. For a 20 μl incubation system, 20 fmol bio-probes and 10 μg protein were obtained from a nuclear extract prepared from DMOG-treated cells. In the competitive binding experiment, the unbiotinylated probes at 5-, 10-, or 100-fold molar excess over the labeled probes were used to challenge the complexes of wild-type probes and proteins. EMSA was performed in accordance with the manufacturer’s instructions of the Light Shift Chemiluminescent EMSA kit (Thermo Fisher Scientific, USA).

### Statistical analysis

All data analyses were carried out using SAS (v9.3). For all analyses, significance was set at the .05 threshold (*p < .05; **p < .01; ns represent not significant).

## Results

### Hypoxia induces the outbreak of ISKNV disease

**Figure 1.**
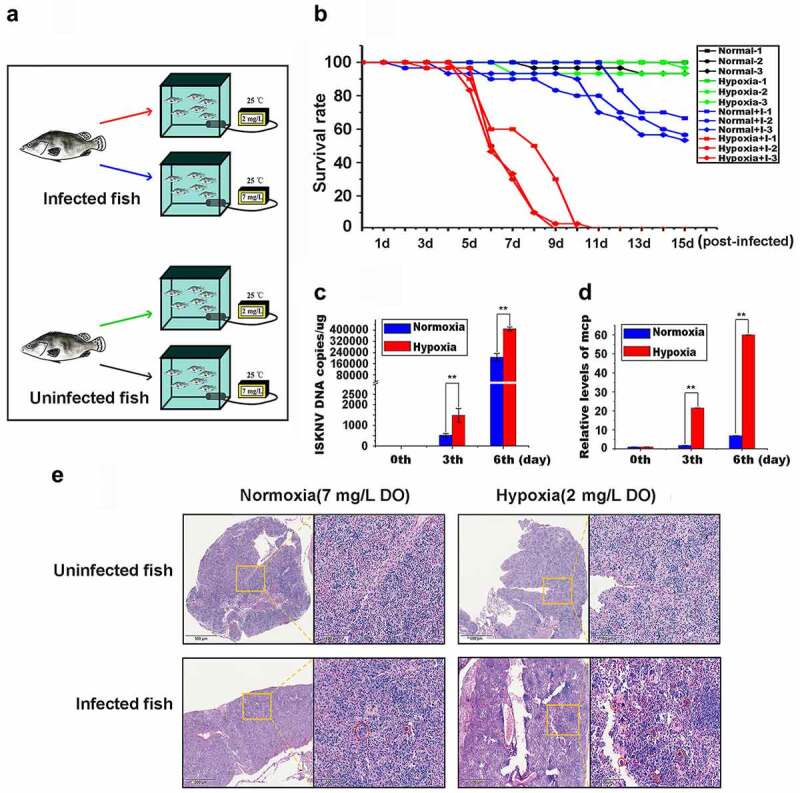
Hypoxia induces the outbreak of ISKNV disease. (A) Infected fish were divided into hypoxia (2 mg/l DO) and normoxia (7 mg/l DO) groups with 30 fish samples for each group. Both groups were cultivated in the experimental tank system setup. as controls, the uninfected fish were cultivated in the same experimental setup. This experimental was repeated thrice. (B) Red line represents infected fish cultured under hypoxia, and blue line denotes infected fish cultured under normoxia. Green line indicates uninfected fish cultured under hypoxic conditions, and black line signifies uninfected fish cultured under normoxia. (C and D) Spleen samples were separated from the mandarin fish at days 0, 3, and 6 p.I. Levels of viral genomic DNA were detected via qPCR, and those of *isknv-mcp* mRNA were detected via qRT-PCR. (E) Spleen samples were separated from the mandarin fish at 6th day p.I. and then used for biopsy and HE staining.

The roles of hypoxia in ISKNV disease outbreaks were identified by establishing a simulation system of hypoxia (Figure 1). Infected fish samples were divided into hypoxia (2 mg/L DO) and normoxia (7 mg/L DO) groups, whereas uninfected fish samples were used as controls. The survival rate of infected fish in the hypoxia (red line) decreased to ~50% at 5–8 days post-infection (p.i.), and all infected fishes died on day 10 p.i. (Figure 1). By comparison, the survival rate of infected fishes in the normoxia (blue line) decreased to 50%–60% on the 15th day p.i., whereas the survival of all uninfected fishes in hypoxic (green line) and normoxic aquariums (black line) was 96.6%–100%. Considering that cell swelling in the spleen is a typical sign of ISKNV infection in mandarin fish [28], the cell morphologies of spleen samples from infected fishes on the 6th day p.i. were further observed via histopathological analysis. The number of swelling cells in the hypoxia group was substantially higher than that in the normoxia group, but no swelling cells were observed in the control groups (Figure 1). Therefore, hypoxia accelerated the outbreak of ISKNV disease. ISKNV replication rate was determined to explore whether hypoxia promotes viral replication to accelerate the outbreak of ISKNV disease. The levels of viral genomic DNA (Figure 1) and *isknv-mcp* mRNA (Figure 1) on the 3rd and 6th days p.i. in the hypoxia group were considerably higher than those in the normoxic group (*p* < .01).

**Figure 2.**
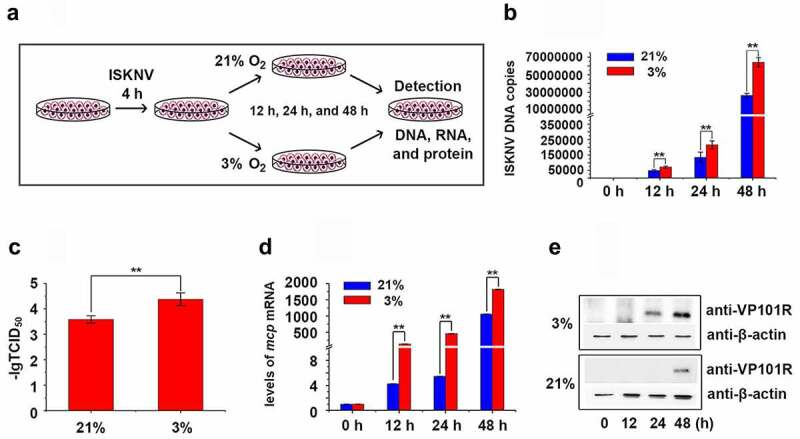
Hypoxia promotes ISKNV replication. (A) Infected cells cultured under hypoxia (3% O_2_) or normoxia (21% O_2_). at 0, 12, 24, and 48 h p.i., the total DNA, RNA, protein, and cell lysates were acquired repeated freezing and thawing for three times and isolated for further analyses. (B) Levels of viral genomic DNA were detected via qPCR. (C) Virus titers (TCID_50_) of the cell lysates acquired by repeated freezing and thawing for three times. (D) Levels of *isknv-mcp* mRNA were detected via qRT-PCR. (E) Levels of ISKNV VP101R were detected via Western blot.

These observations were further confirmed *in vitro*. Infected MFF-1 cells were cultured under hypoxic (3% O_2_) and normoxic conditions (21% O_2_, Figure 2). The expression level of HIF-1α was detected and has no obviously changed at 24 hours and was increased at 48 hours after infected cells under hypoxic (3% O_2_) or normoxic conditions (21% O_2_) (Fig. S2A). The levels of viral genomic DNA, viral titers (TCID_50_ values), *isknv-mcp* mRNA, and ISKNV viral structural protein (VP101R) at 24 and 48 h p.i. in the hypoxia group were remarkably higher than those in the normoxia group (Figure 2). Therefore, hypoxic environments could promote ISKNV replication and trigger the outbreak of ISKNV disease.

### The activated HIF pathway promotes ISKNV replication

**Figure 3.**
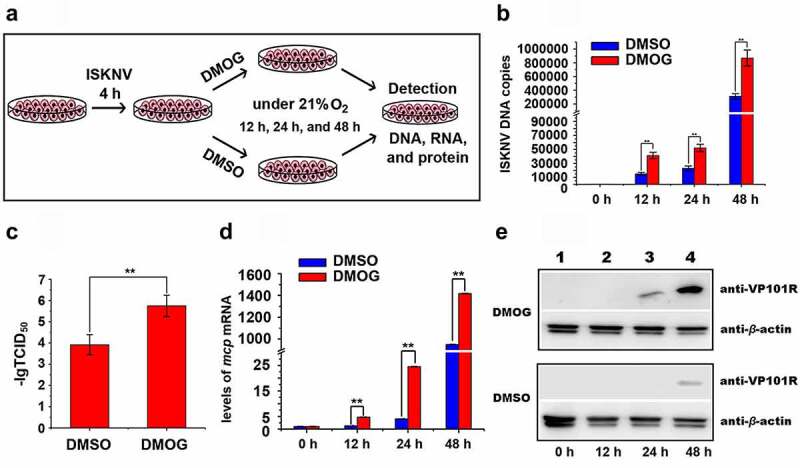
Active HIF pathway promotes ISKNV replication. (A) Infected cells cultured normoxic (21% O_2_) were treated with 1 mM DMOG. at 0, 12, 24, and 48 h p.i., total DNA, RNA, protein, and cell lysates were acquired via repeated freezing and thawing for three times and isolated for further analyses. (B) Levels of viral genomic DNA were detected via qPCR. (C) Virus titers (TCID_50_) of the cell lysates acquired by repeated freezing and thawing. (D) Levels of *isknv-mcp* mRNA were detected via qRT-PCR. (E) Levels of ISKNV VP101R were detected via Western blot.

The HIF pathway mainly regulates the cellular responses to hypoxia stress in mandarin fish [29]. Whether the activated HIF pathway promotes ISKNV replication were investigated. Herein, DMOG was used to activate the HIF pathway [30]. The cells were infected with ISKNV and then treated with 1 mM DMOG. DMSO-treated cells were used as controls (Figure 3). The expression level of HIF-1α was detected and expression level of HIF-1α had no obviously change at 24–48 h after infected cells were treated with DMOG (Fig. S2B). The levels of viral genomic DNA, viral titers, *isknv-mcp* mRNA, and VP101R were determined at indicated times. The levels of viral genomic DNA, viral titers (TCID_50_ values), *isknv-mcp* gene expression, and VP101R in the cells treated with DMOG were remarkably higher than those in the DMSO-treated cells (control group, Figure 3, *p* < .01). Therefore, the activation of HIF pathway promotes ISKNV replication.

### ISKNV encode HREs in the genomic

**Figure 4.**
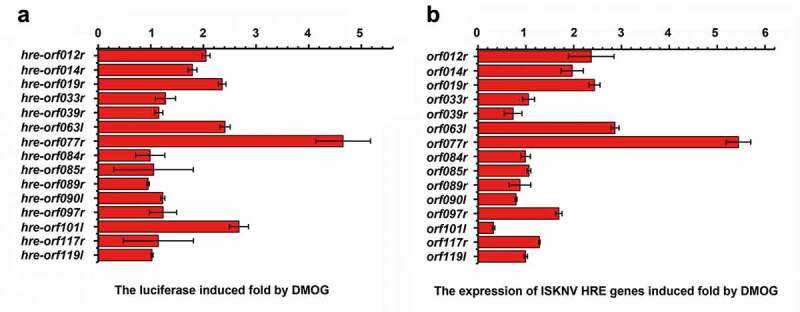
ISKNV HRE-regulated genes respond to the HIF pathway. (A) Cells were co-transfected with 15 predicted pGL3-HREs-luc and pRT-TK plasmids. at 24-h post transfection, dual reporter assay screened the effects of DMOG on all 15 predicted ISKNV HREs in MFF-1 cells. the DMSO-treated cells were used as control. the *y*-axis represents the luciferase treated withDMOG/luciferase treated with DMSO. (B) MFF-1 cells infected with ISKNV. the cells cultured under normoxic (21% O_2_) condition were treated with 1 mM DMOG. the DMSO-treated cells were used as control. at 48 h p.i., total RNAs were acquired for further analyses. Levels of *isknv orf012r, orf014r, orf019r, orf033r, orf039r, orf063l, orf077r, orf084l, orf085r, orf089r, orf090l, orf097r, orf101l, orf117r*, and *orf119l*. mRNA was detected via qRT-PCR. the *y*-axis represents the relative expression levels of *isknv* genes after DMOG treatment/relative expression levels of *isknv* genes after DMSO treatment.

To further investigate whether activation of HIF pathway induces the expression of ISKNV viral genes, we analyzed the HREs 5’-[A/G]CGTG-3’) in the ISKNV genome by using a position weight matrix. The sequences located between −1 and −2,000 bp upstream of the open reading frame (ORF) of viral genes were analyzed. The viral genes were defined as HRE-regulated genes when the upstream of the viral genes contain two or more HREs. As shown in Table 1, ISKNV contained 15 viral HRE-regulated genes, namely, *orf012r, orf014r, orf019r, orf033r, orf039r, orf063l, orf077r, orf084l, orf085r, orf089r, orf090l, orf097r, orf101l, orf117r*, and *orf119l*. Whether the ISKNV viral HREs respond to the HIF pathway was investigated. The activities of HREs-Luc reporter genes were induced by DMOG, an activator of HIF pathway. As shown in Figure 4, the relative luciferase activities (RLAs) of *hre-orf012r, -orf019r, -orf063l, -orf077r*, and *-orf101l* were remarkably upregulated (fold change ≥2), and the highest upregulation was observed for *hre-orf077r* (4.6-fold). Furthermore, the activities of 15 ISKNV viral HRE-regulated genes expression level were analyzed after 48 h of treatment with DMOG. As shown in Figure 4, the expression levels of *orf012r, orf019r, orf063l, and orf077r* were significantly induced (fold change ≥2), and the highest upregulation was observed for *orf077r* (5.4-fold). Therefore, ISKNV encodes HREs in the genome, and some of those viral HREs can respond to the HIF pathway, indicating that the HIF-pathway may induce the expression of ISKNV viral genes.

**Table 1.** Predicted ISKNV-HREs in the ISKNV genome (NC_003494.1)

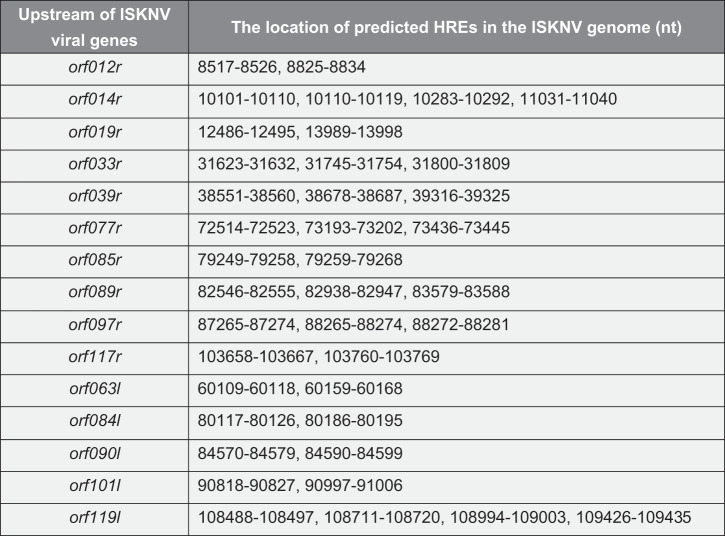

### ISKNV HRE-regulated genes respond to HIF pathway

**Figure 5.**
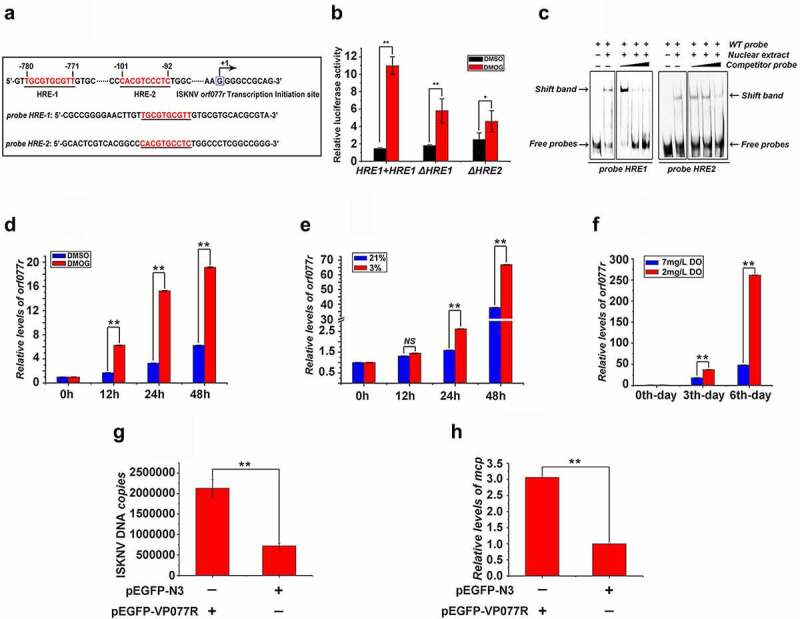
ISKNV HRE-regulated genes respond to HIF pathway. (A) Potential HIF binding sites in the promoter regions of ISKNV *orf077r*. Two putative HIF binding site motifs were identified (HRE-1 and HRE-2). the sequences of a double-stranded oligonucleotide probe containing the HRE-1 and HRE-2 were used for EMSA. (B) MFF-1 cells were transfected with pGL3-*orf077r* HREs-luc, pGL3-*orf077r* HREs-ΔHRE-1 or pGL3-*orf077r* HREs-ΔHRE-2, and the pRT-TK plasmids were co-transfected as inner control. Effects of DMOG on the promoter activities of full length *orf077r* HREs, *orf077r* HREs-ΔHRE-1, and *orf077r* HREs-ΔHRE-2 in MFF-1 cells as detected by dual reporter assay. (C) EMSA assay confirmed that the HIF-1α protein binds to HRE-1 and HRE-2. Cells expressing HIF-1α-GFP. at 24 h post transfection, DMOG was used to activate the HIF-1 pathway, and nuclear proteins were extracted using EMSA assay after 12 h of DMOG treatment. (D) Active HIF pathway induced the expression of *orf077r in vitro*. Infected cells cultured normoxic (21% O_2_) condition were treated with 1 mM DMOG. at 0, 12, 24, and 48 h p.I., total RNAs were acquired via repeated freezing and thawing for three times and isolated for further analyses. Levels of *orf077r* mRNA were detected via qRT-PCR. (E) Hypoxia induces the expression of *orf077r in vitro*. Cells were infected with ISKNV and then cultured under hypoxia (3% O_2_) or normoxia (21% O_2_). at 0, 12, 24, and 48 h p.i., total RNAs were acquired via repeated freezing and thawing for three times and isolated for further analyses. Levels of *orf077r* mRNA were detected via qRT-PCR. (F) Hypoxia induces the expression of *orf077r in vivo*. Mandarin fish were infected with ISKNV and then cultured under hypoxia (2 mg/l DO) and normoxia (7 mg/l DO). at 0, 3, and 6 days p.i., total RNAs were acquired via repeated freezing and thawing for three times and isolated for further analyses. Levels of *orf077r* mRNA were detected via qRT-PCR. (G-H) Cells expressing VP077R-GFP were infected with ISKNV. at 48 h p.i., total DNA and RNA were acquired. Levels of viral genomic DNA were detected via absolute qPCR. Levels of *isknv-mcp* mRNA were detected via qRT-PCR.

As a notable response to the HIF pathway, the ISKNV *orf077r* was chosen as an example to confirm that HIF pathway induced the expression of ISKNV HREs containing viral genes. *Hre-orf077r was* chosen as an example to reveal how HIF-1α/HIF-1β heterodimers bind to ISKNV viral HREs and are regulated by HIF pathway. The activities of HRE-1 and HRE-2 (Figure 5) between −1 and −2,000 bp upstream of the transcription start site of *orf077r* full-length cDNA were determined. The RLA of the *orf077r* promoter (containing the HRE-1 and HRE-2) increased by 7.5-fold after the cells were treated with DMOG, whereas the RLAs of mutant promoters (ΔHRE-1 and ΔHRE-2) increased by only 3.6- and 1.7-fold (Figure 5). The binding of HIF to the HREs of *orf077r* was explored via EMSA assay (Figure 5). The band shifts of protein–DNA complexes were detected when nucleoproteins, which were separated from cells treated with DMOG after overexpressing mandarin fish HIF-1α [29], were incubated with biotin-labeled HRE-1 or HRE-2 probes. Meanwhile, competitively reduced band shifts were observed when the nucleoproteins were incubated with unlabeled HRE-1 or HRE-2 probes at a 5-, 10-, and 100-fold molar excess. To further investigate whether the expression of *orf077r* response the HIF pathway active, the level of *orf077r* mRNA was detected. As shown in Figure 5, the level of *orf077r* mRNA dramatically increased after the infected cells were treated with DMOG or the infected cells/fish were subjected to hypoxic conditions. Therefore, ISKNV viral *hre-orf077r* could bind with HIF-1α, and their regulated viral genes respond to the HIF pathway. Whether *orf077r* genes could promote he ISKNV replication was also investigated. The levels of viral genomic DNA and *isknv-mcp* mRNA increased after *orf077r* overexpression (Figure 5). Therefore, *orf077r* could promote ISKNV replication, and the hypoxia could promote ISKNV replication possibly by inducing HRE activities.

### Frequencies of viral HREs among 16 families of aquatic animal virus

**Figure 6.**
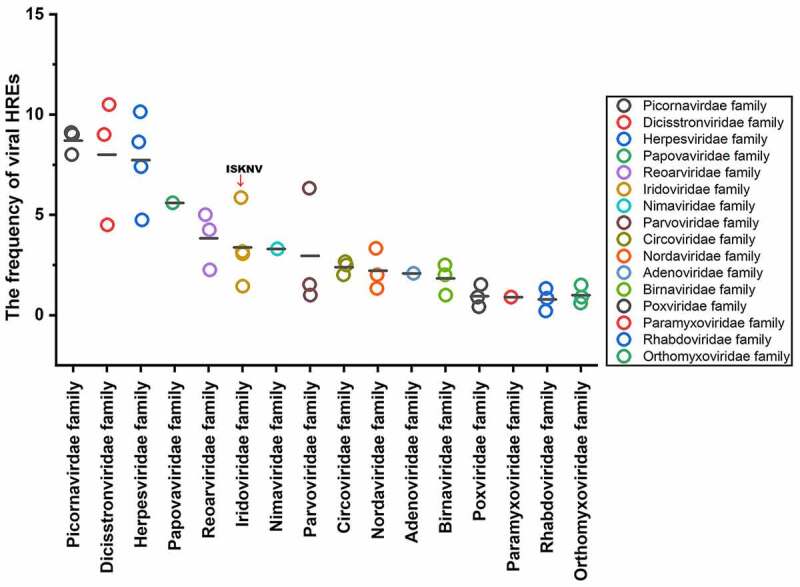
Frequency of HREs in aquatic animal viruses from 16 families. HRE frequency of each virus: carp picornavirus 1 (9.0), clownfish picornavirus (9.0), bluegill picornavirus (8.0), mud crab dicistrovirus (4.5), *Macrobrachium rosenbergii* Taihu virus (9.0), taura syndrome virus (10.5), abalone herpesvirus (10.14), cyprinid herpesvirus 3 (8.63), anguillid herpesvirus 1 (7.4), ictalurid herpesvirus 2 (4.74), marbled eel polyomavirus (5.6), grass carp reovirus (5.0), *Scylla serrata* reovirus (2.25), piscine reovirus (4.25), infectious spleen and kidney necrosis virus (5.86), Singapore grouper iridovirus (3.05), lymphocystis disease virus 1 (1.44), frog virus 3 (3.16), white spot syndrome virus (3.3), tilapia parvovirus (6.33), infectious hypodermal and hematopoietic necrosis virus (1.0), sea star-associated densovirus (1.53), clinch densovirus 1 (2.8), uncultured virus clone AfaCV3 (2), uncultured virus clone SdaCV2 (2.5), *Anguilla anguilla* circovirus (2.66), red-spotted grouper nervous necrosis virus (3.33); tiger puffer nervous necrosis virus (2.0); covert mortality nodavirus (1.33), white sturgeon adenovirus 1 (2.08), spring viremia of carp virus (.2), viral hemorrhagic septicemia virus (.83), hirame rhabdovirus (1.33), infectious salmon anemia virus (.2), Tilapia lake virus (.9), pilchard orthomyxovirus (1.5), carp edema virus (.41), salmon gill poxvirus (1.53), cheloniid poxvirus 1 (.9), Atlantic salmon paramyxovirus (.89), infectious pancreatic necrosis virus (1), Tasmanian aquabirnavirus (2), and Tellina virus 1 (2.5).

The frequencies of viral HREs relative to the number of viral ORFs were used to represent the sensitivity of viruses to HIF pathway [27]. Analysis was conducted on 43 species aquatic animal viruses from 16 families (Table S1), whose host ranged among the amphibians, vertebrates, echinoderms, or crustaceans, including families *Picornavirdea, Dicistronviridae, Herpesviridae, Papovaviridae, Reoviridae, Iridoviridae, Nimaviridae, Parvoviridae, Circoviridae, Nordaviridae, Adenoviridae, Rhabdoviridae, Orthomyxoviridae, Paramyxoviridae, Birnaviridae*, and *Orthomyxoviridae*. As shown in Figure 6, the viral HRE frequencies of the aquatic animal viruses tested ranged from .2 to 10.5. The viral HRE frequency of ISKNV (family *Iridoviridae*) was 5.86, which is above the average frequency of HREs among these aquatic animal viruses. Families*Picornavirdea, Dicistronviridae*, and *Herpesviridae* had a high frequency of HREs (higher than ISKNV). Therefore, families *Picornavirdea, Dicistronviridae*, and *Herpesviridae* might be more sensitive to aquatic environment hypoxia stress compared with the other species.

## Discussion

The “epidemiologic triad” is an important conceptual model used to understand the causes of diseases [31]. For diseases to emerge, the host must be genetically susceptible, the pathogens must be genetically virulent, and the environment must favor disease outbreaks. In mammals, changed environmental conditions modulate the pathogens’ virulence and host susceptibility, thereby influencing the rate and pattern of epidemics [32–34]. Cold-blooded vertebrates (reptiles, amphibians and fish) are susceptible to environmental changes which regulate cell biology, pathophysiologic processes, and viral infections. Environmental factors, such as water temperature, salinity, nitrogen compounds, and dissolved oxygen, play an important role in the outbreak of viral diseases in cold-blooded vertebrates [35–37]. Hypoxia has been reported to be related to bacterial disease outbreaks in aquatic environments, for example, the mortality of tilapia (*Oreochromis* sp.) infected with *Streptococcus* sp. remarkably increases under hypoxia [38], and low DO levels increased the mortality of yellowtail jacks (*Seriola quinqueradiata*) infected with *Enterococcus seriolicida* [39]. However, the phenomenon of hypoxia influencing the outbreak of viral disease in cold-blooded vertebrates remains unclear. Our results showed that hypoxic environments could promote ISKNV replication and trigger the outbreak of ISKNV disease. Thus, hypoxia increases the risk of viral disease outbreaks in cold-blooded vertebrates.

**Figure 7.**
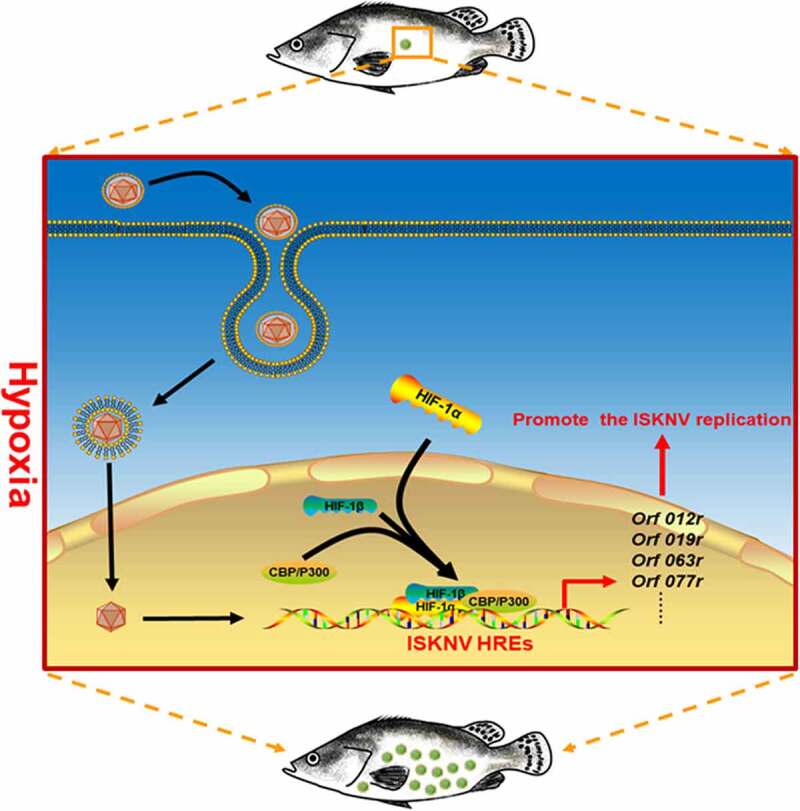
Mechanism of hypoxia trigger the outbreak of ISKNV disease. When the mandarin fish was subjected to hypoxia, the HIF pathway was activated. Then, the activated HIF pathway promoted the ISKNV viral gene expression through the functional HREs leading to promoter ISKNV replication.

The mechanism by which hypoxia triggers ISKNV disease outbreaks was elucidated in this work. Many studies have indicated that hypoxia stress may influence the host immunity, indirectly leading to lowered host resistance to pathogen infections [40]. In this study, we focused on whether hypoxia directly participates in the outbreaks of ISKNV disease. Results suggested that the hypoxia/active HIF pathway significantly upregulated the expression levels of viral HREs genes (e.g., *orf012r, orf019r, orf063l, and orf077r*). To investigate whether ISKNV via HREs benefits viral replications, the ISKNV *orf077r* gene, which was identified as a major viral HRE-regulated gene, was selected for detailed analysis. Our results showed that HIF-1α could directly bind to the *hre-orf077r* promoter. The hypoxia/HIF pathway could strongly induce *orf077r* expression, and the overexpression *orf077r* could promote ISKNV replication. These results implied a potential mechanism of hypoxia directly triggering the outbreak of ISKNV disease. Hypoxia activated the HIF pathway and promoted the expression of the viral functional HRE genes, leading to enhanced ISKNV replication and triggering the ISKNV disease outbreak (Figure 7).

The expansion of a hypoxic area in aquatic ecosystems has become a major concern over the past few decades [41–43]. However, the outbreak of viral diseases in aquatic animal under hypoxia, an important aspect of the negative effect of aquatic hypoxia, is rarely evaluated. In this study, the frequencies of viral HREs in the viral genome among 16 families of aquatic animal viruses, whose host ranged among the amphibians, vertebrates, echinoderms, or crustaceans, were analyzed and showed an approximately 50-fold variation. Some viruses (such as *Picornavirdea, Dicistronviridae*, and *Herpesviridae*) had a relatively high frequency of HREs, indicating that these viral diseases may prevail when the hypoxic area expands in aquatic ecosystems. What’s more, HREs were also present in the promoter area of important viral genes in aquaculture animal viruses. For example, grass carp reovirus component protein VP4 plays a key role in viral genome transcription and replication [44], the HREs were in the viral genome segment 5 which encoded VP4; cyprinid herpesvirus 3 genes possibly involved in DNA replication (*orf47* and *orf71* [45]) and nucleotide metabolism (*orf19* and *orf23* [45]) have HREs in the gene promoter region; White spot syndrome virus encodes HREs in front of some immediate early genes (wsv056, wsv100, wsv249 and wsv403 [46]). These findings implied that hypoxia might induce the expression of those viral genes to trigger viral disease outbreaks. Our work will help in predicting viral disease outbreaks and developing intervention strategies for viral disease control when encountering hypoxia during aquaculture and aquatic ecological protection.

## Supplementary Material

Supplemental Material

## Data Availability

The authors confirm that the data supporting the findings of this study are available within the article and its supplementary materials.
