# Supplementary material for: Hypoxia triggers the outbreak of infectious spleen and kidney necrosis virus disease through viral hypoxia response elements
Source: Virulence. 2022 Apr 25;13(1):714–26. doi: 10.1080/21505594.2022.2065950 (PMC9045828; doi:10.1080/21505594.2022.2065950)
Supplement: Supplemental Material [file KVIR_A_2065950_SM4448.zip › supplementary/Table S1.docx]

**Table S1: The viral genomic used to hypoxia response element predict**

| **Virus family name** | **Gen Bank ID** | | | |
| --- | --- | --- | --- | --- |
| ***Picornavirdea*** | Carp picornavirus 1  (NC_023162.1) | Clownfish picornavirus（MT080322.1） | Bluegill picornavirus  (NC_018506.1) |  |
| ***Dicistronviridae*** | Mud crab dicistrovirus (HM777507.1) | Macrobrachium rosenbergii Taihu virus  (NC_018570) | Taura syndrome virus  (MT877008.1) |  |
| ***Herpesviridae*** | Cyprinid herpesvirus 3  (NC_009127） | Anguillid herpesvirus 1 (NC_013668) | Ictalurid herpesvirus 2  ( MG271984.1) | Abalone Herpesvirus (JF967012.2) |
| ***Papovaviridae*** | Marbled eel polyomavirus （KX781210.1） |  |  |  |
| ***Reoarviridae*** | Grass carp reovirus  (KR180368.1-KR180378.1) | Scylla serrata reovirus  (HQ414127.1-HQ414138.1 ) | Piscine reovirus (NC_036468.1-NC_036477.1) |  |
| ***iridoviridae*** | Infectious spleen and kidney necrosis virus （NC_003494.1） | Singapore grouper iridovirus (NC_006549.1) | Lymphocystis disease virus 1（NC_001824） | Frog virus 3（NC_005946.1） |
| ***Nimaviridae*** | White spot syndrome virus  (KT995472.1) |  |  |  |
| ***Parvoviridae*** | Tilapia parvovirus  (MW685502.1) | Infectious hypodermal and hematopoietic necrosis virus  (NC_002190 ) | Sea star-associated densovirus (NC_038532.1) | Clinch densovirus 1（MT341473.1） |
| ***Circoviridae*** | AfaCV3（KX246255.1） | SdaCV2（KX246259.1） | Anguilla anguilla circovirus  (KC469701) |  |
| ***Nordaviridae*** | Tiger puffer nervous necrosis virus  (NC_013460.1-NC_013461.1) | Covert mortality nodavirus  (MT270123.1-MT270124.1) | Redspotted grouper nervous necrosis virus  (NC_008040.1 -NC_008041.1) |  |
| ***Adenoviridae*** | White sturgeon adenovirus 1（MK101347.1） |  |  |  |
| ***Rhabdoviridae*** | Spring viremia of carp virus  (U18101.2) | Viral hemorrhagic septicemia virus  (MK829686.1) | Hirame rhabdovirus  (NC_005093.1) |  |
| ***Orthomyxoviridae*** | Infectious salmon anemia virus  (NC_006497.1-NC_006505.1) | Tilapia lake virus (NC_029921.1 -NC_029930.1) | Pilchard orthomyxovirus  (MN241407.1- MN241414.1) |  |
| ***Poxviridae*** | Carp edema virus  (LC613089.1) | Salmon gill poxvirus (NC_027707.1) | Cheloniid poxvirus 1 (MT799800.1) |  |
| ***Paramyxoviridae*** | Atlantic salmon paramyxovirus （NC_025360.1） |  |  |  |
| ***Birnaviridae*** | Infectious pancreatic necrosis virus (NC_001915.1-NC_001916.1) | Tasmanian aquabirnavirus (NC_028252.1- NC_028253.1) | Tellina virus 1（NC_038869.1- NC_038870.1） |  |
