# Supplementary material for: Hypoxia triggers the outbreak of infectious spleen and kidney necrosis virus disease through viral hypoxia response elements
Source: Virulence. 2022 Apr 25;13(1):714–26. doi: 10.1080/21505594.2022.2065950 (PMC9045828; doi:10.1080/21505594.2022.2065950)
Supplement: Supplemental Material [file KVIR_A_2065950_SM4448.zip › supplementary/Table S2.docx]

**Table S2: Primers used in this paper**

| **Primer** | **Sequence （5’-3’）** | **Reference** |
| --- | --- | --- |
| pEGFP-N3-*orf*077R (F) | GGAATTCTATGGATTCCCTTGTCGACCT | This study |
| pEGFP-N3-*orf*077R (R) | GGGGTACCTAGTGTTTTATATACCCCAAAG | This study |
| pEGFP-N3-HIF-1α (F) | GGAATTCTATGGACACAGGAATTGTACCAGAAAAG | This study |
| pEGFP-N3-HIF-1α (R) | GGGGTACCGTTGACGTGGTCCAGAGC | This study |
| pGL3-*orf012r* (F) | TCCCCCGGGAGAGTGTCATGGGTGTGGTG | This study |
| pGL3-*orf012r* (R) | GAAGATCTATGACAGAGCTTATGGCGGCGTC | This study |
| pGL3-*orf014r* (F) | GGGGTACCTAGACAAGCGGCGCATTATACACAATTA | This study |
| pGL3-*orf014r* (R) | CCCTCGAGTGCGCCGCAGTGTAAAGGCAT | This study |
| pGL3-*orf019r* (F) | TCCCCCGGGGCCAGAGCCCATACTTATCCCATG | This study |
| pGL3-*orf019r* (R) | GAAGATCTGTACTACACATTACACAAGATGCGCA | This study |
| pGL3-*orf033l* (F) | GGGGTACCAACAACGCTGTGGGTTTCATTGTA | This study |
| pGL3-*orf033l* (R) | CCCTCGAGTACGTGTGCGGTGATGAGGGTG | This study |
| pGL3-*orf039r* (F) | GGGGTACCTGGGAGACTGTGCTGTTTATCTGACTCGCC | This study |
| pGL3-*orf039r* (R) | CCCTCGAGGGTGCGGTATATGTTTTTTACACC | This study |
| pGL3-*orf077r* (F) | GGGGTACCAATGGCCCTTGTGGTTAGGC | This study |
| pGL3-*orf077r* (R) | CCTAAGCTTCATTTTTATTACCAAAAAACATGTTAG | This study |
| Overlap-*orf077r*-ΔHRE-1 (F) | ACATACGCGTGCACGCACACAAGTTCCCCGGCGTGC | This study |
| Overlap-*orf077r*-ΔHRE-1 (R) | GCACGCCGGGGAACTTGTGTGCGTGCACGCGTATGT | This study |
| Overlap-*orf077r*-ΔHRE-2 (F) | CATCCCGGCCGAGGGCCAGGCCGTGACGAGTGCATC | This study |
| Overlap-*orf077r*-ΔHRE-2 (R) | GATGCACTCGTCACGGCCTGGCCCTCGGCCGGGATG | This study |
| pGL3-*orf085r* (F) | GGGGTACCACAGCATTATGTCTGCCGAGAC | This study |
| pGL3-*orf085r* (R) | CCCTCGAGACATCGTTTTGGCCTGTGGACAC | This study |
| pGL3-*orf089r* (F) | GGGGTACCTGCGCATAGCGTGCGGACAGTACCCGCA | This study |
| pGL3-*orf089r* (R) | CCCTCGAGTCTTGGTTATTGCCGCATTTGCCTA | This study |
| pGL3-*orf097r* (F) | CTAGCTAGCCAACGGTAGATGGCATCGGTG | This study |
| pGL3-*orf097r* (R) | GAAGATCTGTGCGCACACAACATAACACTTTGTGTAG | This study |
| pGL3-*orf117r* (F) | GGGGTACCATTCAAAAACGTTGCGCCTTCG | This study |
| pGL3-*orf117r* (R) | CCCTCGAGGCGGTGAACTGCAGACTGTC | This study |
| pGL3-*orf063l* (F) | GGGGTACCCCGTCACCCTGGTGTTGGCCT | This study |
| pGL3-*orf063l* (R) | GAAGATCTTCCATTGTACATACGGCTTCAATCGCACT | This study |
| pGL3-*orf084l* (F) | GGGGTACCCTAAGTGTTCGTGCTCCAGTCATCAG | This study |
| pGL3-*orf084l* (R) | CCCTCGAGATGCGCGGTCCATCTCACAGCA | This study |
| pGL3-*orf090l* (F) | GGGGTACCGTCGGCGCACGGTGAATACCAG | This study |
| pGL3-*orf090l* (R) | CCCTCGAGGGGCGTATACACAGCAACCAACAC | This study |
| pGL3-*orf101l* (F) | GGGGTACCTTGGTTTTGATGATAGCTTTGACCATGTTG | This study |
| pGL3-*orf101l* (R) | CCCTCGAGCATGTACTCGTCATAGCATATAGTTACAAG | This study |
| pGL3-*orf119l* (F) | GGGGTACCTGGTGTAAGGCCATCTGGCAGGAT | This study |
| pGL3-*orf119l* (R) | CCCTCGAGCGGCTCCTATGGGCCATACG | This study |
| RT-qPCR-*orf012r* (F) | CAAACGGTACGGTCTGGTCAATACA | This study |
| RT-qPCR-*orf012r* (R) | CACCACAATGTTGAACGGCACTC | This study |
| RT-qPCR-*orf014r* (F) | TAACGAGGTCTATGTTGTCGTCCAAA | This study |
| RT-qPCR-*orf014r* (R) | GCGTGCGCCGCAGTGTAA | This study |
| RT-qPCR-*orf019r* (F) | CTACGGCATAGCCCCAAACAAC | This study |
| RT-qPCR-*orf019r* (R) | GTGCTGGGTGAATGTGGAACG | This study |
| RT-qPCR-*orf033r* (F) | TGTGGACGCCAGCGGTATG | This study |
| RT-qPCR-*orf033r* (R) | CGGCCCCAGTCGTCTTTGTA | This study |
| RT-qPCR-*orf039r* (F) | CACCGACGACACCGACCGA | This study |
| RT-qPCR-*orf039r* (R) | CCGCCGCAGCACTCACATC | This study |
| RT-qPCR-*orf063l* (F) | GCCCCGAAACGCCATACAA | This study |
| RT-qPCR-*orf063l* (R) | GACTGAAAGTTCCGCATAAGACCC | This study |
| RT-qPCR-*orf077r* (F) | GAGTACAATGCGGAGGGCTTCA | This study |
| RT-qPCR-*orf077r* (R) | TGGCCATCGTTGGGGTCTG | This study |
| RT-qPCR-*orf084l* (F) | GGATTCTACTCACAAGCACCTGGAC | This study |
| RT-qPCR-*orf084l* (R) | CACAATCGCCTTCCCATCCTCT | This study |
| RT-qPCR-*orf085r* (F) | CACCACCCTCGCCTCTACCC | This study |
| RT-qPCR-*orf085r* (R) | CCTGTCCCTCGCTTCCTCCTG | This study |
| RT-qPCR-*orf089r* (F) | CAACGAGCGCCACCATGACC | This study |
| RT-qPCR-*orf089r* (R) | AGCCCGACCCGCCTAAACC | This study |
| RT-qPCR-*orf090l* (F) | AGCTGCTGCCCAATGCCG | This study |
| RT-qPCR-*orf090l* (R) | TTGTGACAGATCCAGTGTCGATTATTT | This study |
| RT-qPCR-*orf097r* (F) | GCATGACGCCCTTGTCTGTAGC | This study |
| RT-qPCR-*orf097r* (R) | CGGACTTTAAGGAGGTGCTTGTTG | This study |
| RT-qPCR-*orf101l* (F) | AAGCCGAGGACCCCAAGAAGT | This study |
| RT-qPCR-*orf101l* (R) | GTCCTGACCGCCCACCAGTAT | This study |
| RT-qPCR-*orf117r* (F) | ACGTGCAAGCGGTGTCGG | This study |
| RT-qPCR-*orf117r* (R) | CGGTCGGGTGGTTGGAAGTT | This study |
| RT-qPCR-*orf119l* (F) | CAGCCGCACAGCACAACAAC | This study |
| RT-qPCR-*orf119l* (R) | CCGCCAGCAACGCTTTCA | This study |
| RT-qPCR-*mcp* (F) | CAATGTAGCACCCGCACTGACC | This study |
| RT-qPCR-*mcp* (R) | ACCTCACGCTCCTCACTTGTC | This study |
| RT-qPCR-*hif-1α* (F) | CTCTGGAAGGCTTTCTCATGGTG | This study |
| RT-qPCR-*hif-1α* (R) | GGTCACAGGGATGTATGAAGTCAA | This study |
| RT-qPCR-*β-actin*(F) | CCCTCTGAACCCCAAAGCCA | This study |
| RT-qPCR-*β-actin*(R) | CAGCCTGGATGGCAACGTACA | This study |
