# Supplementary figures and images for: Hypoxia triggers the outbreak of infectious spleen and kidney necrosis virus disease through viral hypoxia response elements
Source: Virulence. 2022 Apr 25;13(1):714–26. doi: 10.1080/21505594.2022.2065950 (PMC9045828; doi:10.1080/21505594.2022.2065950)

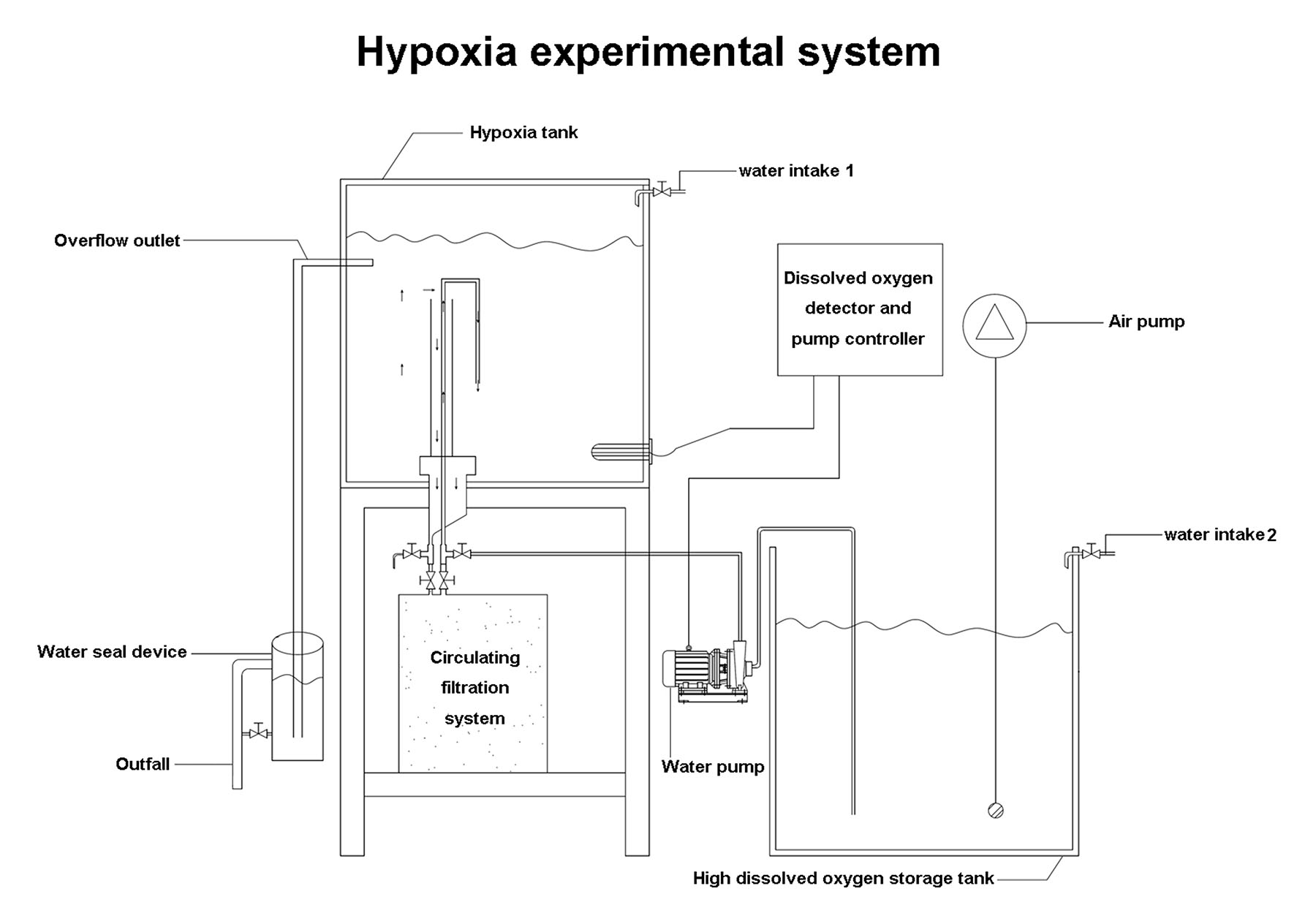

Supplement: Supplemental Material [file KVIR_A_2065950_SM4448.zip › supplementary/Figure. S1.jpg]

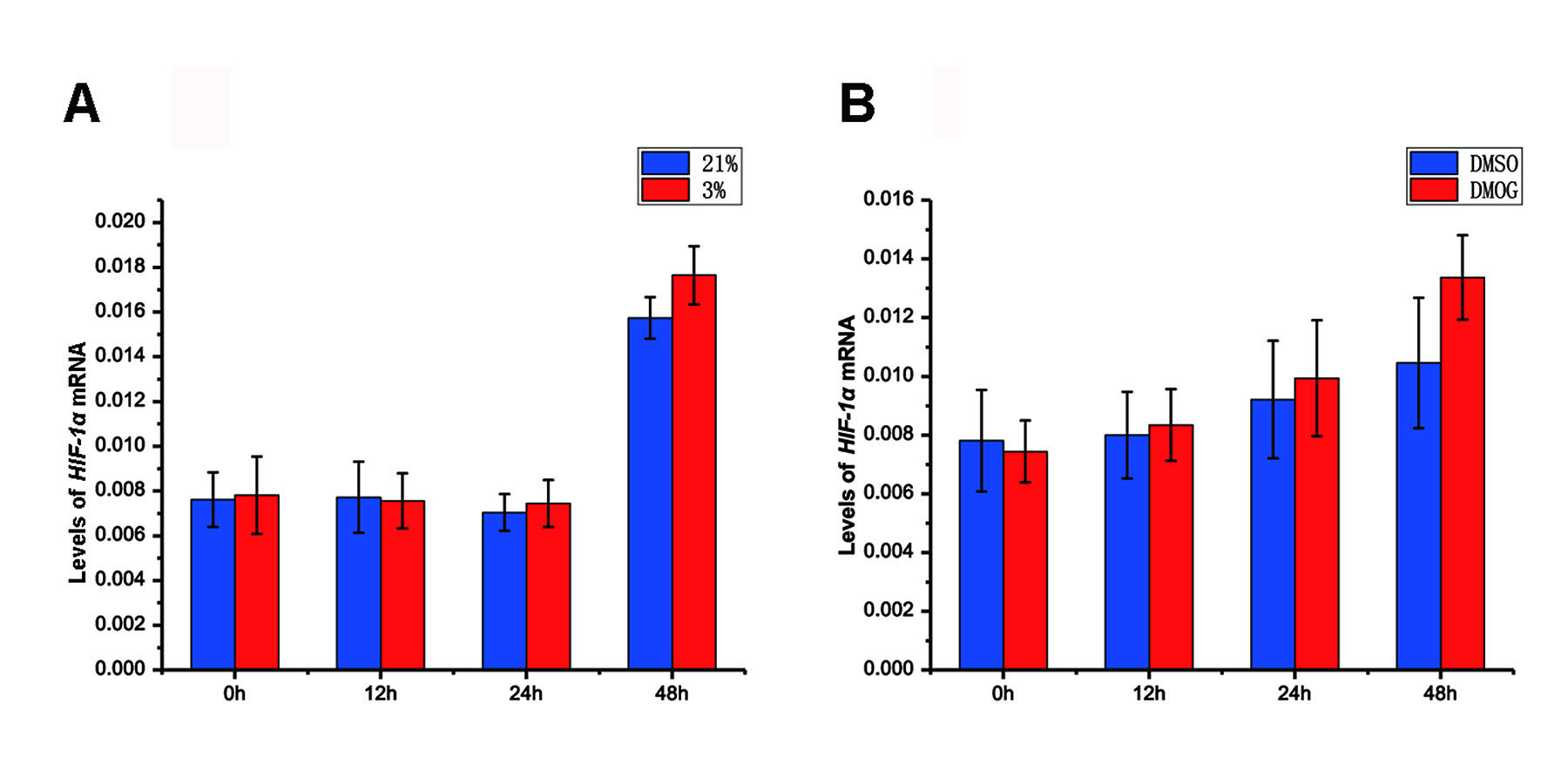

Supplement: Supplemental Material [file KVIR_A_2065950_SM4448.zip › supplementary/Figure. S2.jpg]
